# Out-Smarting the Host: Bacteria Maneuvering the Immune Response to Favor Their Survival

**DOI:** 10.3389/fimmu.2020.00819

**Published:** 2020-05-12

**Authors:** Nastaran Mues, Hong Wei Chu

**Affiliations:** Department of Medicine, National Jewish Health, Denver, CO, United States

**Keywords:** toll like receptors, bacterial infection, immune response, airways, inflammation

## Abstract

Bacteria adapt themselves to various environmental conditions in nature, which can lead to bacterial adaptation and persistence in the host as commensals or pathogens. In healthy individuals, host defense mechanisms prevent the opportunistic bacteria/commensals from becoming a pathological infection. However, certain pathological conditions can impair normal defense barriers leading to bacterial survival and persistence. Under pathological conditions such as chronic lung inflammation, bacteria employ various mechanisms from structural changes to protease secretion to manipulate and evade the host immune response and create a niche permitting commensal bacteria to thrive into infections. Therefore, understanding the mechanisms by which pathogenic bacteria survive in the host tissues and organs may offer new strategies to overcome persistent bacterial infections. In this review, we will discuss and highlight the complex interactions between airway pathogenic bacteria and immune responses in several major chronic inflammatory diseases such as asthma and chronic obstructive pulmonary disease (COPD).

## Introduction

Human lungs are in constant interaction with different bacteria and bacterial particles and are exposed to a variety of environmental threats. The healthy human lungs used to be considered sterile, but recent studies have reshaped this belief. In fact, human lungs are colonized with diverse bacteria including the genera *Prevotella, Streptococcus, Klebsiella, Veillonella, Neisseria, Haemophilus, pseudomonas, and Fusobacterium* (1, 2). These bacterial species can particularly persist in the lungs and often give rise to super infections particularly followed by viral infections. Microbes maintain a lower density in a healthy lung (about 10^3^–10^5^ CFU per gram of the tissue) as compared to the gut with a load of 10^11^ CFU per gram of the tissue. It has been shown that in airway chronic inflammatory diseases such as asthma, there is a shift in the lung microbiota toward a greater diversity in species richness (3).

Normal human lung resident cells such as macrophages and epithelial cells employ a complex defense mechanism to cope with the pathogenic infection vs. commensal bacteria and their products. The innate immune response is the first line of defense that protects the lungs from pathogenic microbes and their secreted products. The lung epithelium cells act as a barrier with goblet cells secreting mucus and ciliated cells transporting mucus containing microbes and microbial particles away from the distal lung. In chronic respiratory diseases, such as cystic fibrosis (CF), COPD, and asthma, mucus hypersecretion and dysfunctional ciliated cells can disturb this barrier leading to less to no clearance of the bacteria from the lungs (3–5). Alveolar macrophages act as the primary phagocytes of the innate immunity in the lung. Airway epithelial cells and macrophages also secrete inflammatory cytokines in response to pathogens and their particles (6). Immune system utilizes several pathways such as toll like receptors (TLRs), NOD like receptors (NLRs), and inflammasome to recognize microbial particles and induce the production of antimicrobial proteins and peptides like lyzozymes, defensins and cathelicidines that effectively stop microbial infection (7–9). Some of these antimicrobial peptides like defensins and cathelicidines have chemotactic properties and recruit immune cells like macrophages and neutrophils to the site of infection (10, 11).

Although inflammation is a pivotal response to microbial infections, it may damage the host cells or tissues and create an environment that allows pathogenic bacteria to employ evading mechanisms to outsmart the host for their survival and persistence. The major goal of this review is to present some unique survival mechanisms exploited by several strains of bacteria commonly seen in lung infectious and inflammatory processes related to the utilization of the host TLR signaling pathways. In addition, our review is mainly focused on the evasion of bacterial infection in chronic inflammatory lung diseases, and there is no doubt that these examples only scratch the surface of this forthcoming research area. We foresee that current research is moving toward investigating bacterial infections in specific niche environments of the host, and that these insights discussed here can enhance our perspective in which the pathogen evade the immune system.

## Tlrs Signaling: a Two-Edge Sword in Bacterial Infection of Asthmatic or Allergic Airways

Toll like receptors are a family of highly conserved and pattern recognition receptors (PRRs) that bind to microbial pathogen associated molecular patterns (PAMPs) also called microbial associated molecular patterns (MAMPs). TLRs also bind to endogenous molecules released from the host dying cells known as danger associated molecular pattern (DAMPs). Different TLRs are expressed on different cell types including immune cells and airway epithelial cells and bind to different ligands, which upon activation in healthy individuals can promote an appropriate inflammatory response. TLR ligands consists of, but not limited to, bacterial cell wall components like lipopolysaccharides (LPS) in Gram-negative bacteria and teichoic acid in Gram-positive bacteria, viral double stranded RNA, single stranded or double stranded DNA, flagellin, etc (5, 12). TLRs are transmembrane receptors that contain a leucine rich extracellular domain and a highly conserved Toll interleukin-1 receptor (TIR) domain. So far, there have been 10 human and 12 murine TLRs identified, and each recognizes a specific set of molecular pattern. In humans, TLR1, 2, 4, 5, and 6 reside on the cell membrane, while TLR3, 7, 8, and 9 are located in endosomes, lysosomes, or endoplasmic reticulum (ER). Upon binding to their ligand, TLRs initiate the inflammatory response by activating their target downstream signaling pathways, including the recruitment of adaptor proteins such as myeloid differentiation factor 88 (MyD88) to the TIR domain. MyD88 activates downstream signaling targets including IRAK family kinases and results in activation of transcription factors of nuclear factor-κB (NF-κB), mitogen-activated protein kinases (MAPK), and activator protein-1 (AP-1). These transcription factors facilitate up-regulation of pro-inflammatory cytokines and type I interferons transcription (13).

**FIGURE 1:**
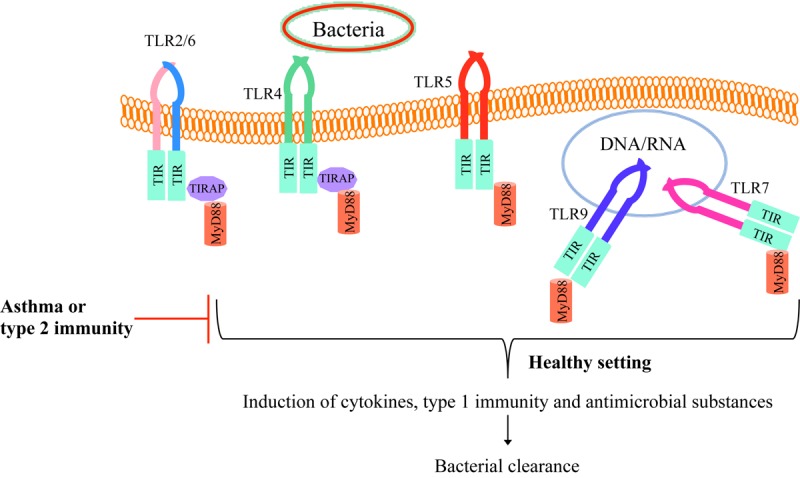
TLR signaling in healthy individuals and asthmatics. Bacteria and their components bind to TLRs on the cell membrane (TLR2/6, TLR4, or TLR5) or enter the cell cytoplasm and bind to endosomal TLRs (TLR 7 or TLR9). Upon binding the bacterial particle to its receptor, TLR recruit MyD88 and subsequently induce host defense cytokines and antimicrobial mediators as well as the type 1 immune response, enhancing bacterial clearance from the tissue (e.g., lung). However, in allergic asthma or type 2 inflammation environment, TLR activity is dampened, which allows the bacteria to survive and hide from the normal defense mechanisms.

Toll like receptors-mediated proper inflammatory response in healthy individuals leads to inflammation induction and bacterial clearance from the lungs. In the field of experimental asthma and allergic airways, the pre-existing type 2 inflammation environment reduces normal TLR function to allow the bacteria to survive and hide from the immune response in part by inhibiting the production of antimicrobial substances (Figure 1). The persistence of bacteria in turn attempts to suppress the type 2 inflammatory response, but it may fail to do so, leading to asthma exacerbations. One of the mechanisms is that bacterial products can hijack the immune system for their benefit by recruiting regulatory T cells (14, 15). Efforts of reducing inflammation can be at the cost of higher risk of opportunistic/commensal bacteria persistence in the lungs as an appropriate pro-inflammatory response is critical to recruit leukocytes such as neutrophils to eliminate pathogens. Collectively, insufficient TLR signaling activation by bacteria in allergic airways and asthma may lead to bacterial survival and persistence.

## Evading Mechanisms of Bacteria in Lung Diseases

Patients with chronic airway inflammatory diseases are generally susceptible to opportunistic bacterial infections that can persist from months or even years. Notably, about more than 50% of COPD infective exacerbations are due to bacterial infections (16). These infections can cause disease exacerbations and promote disease progression. Here, we will discuss how several common bacterial species utilize their tactics to evade the host effective defending mechanisms.

## Non-Typeable *Haemophilus Influenzae*, an Unwanted Guest in Small Airways

Non-typeable *Haemophilus influenzae* (NTHi) is a non-capsulated Gram-negative bacterium, which resides in human nasopharynx as a common commensal. Interestingly, NTHi is a common opportunistic and human exclusive pathogen bacterium of lower respiratory tract and small airways causing chronic and repetitive infections and structural damages especially in COPD patients (17). It has consistently been shown that NTHi can survive in the lungs by attaching and or entering human airway epithelial cells (18–20). NTHi invasion and entry into airway epithelial cells can be through microvilli and lamellipodia extensions of epithelial cells that form a vesicle around the live bacteria and facilitate NTHi entrance into these cells (21). Additionally, the role of actin and tubulin cytoskeletons was confirmed in NTHi internalization since inhibiting actin and tubulin construction with Cytochalasin D and colchicine, constrained NTHi invasion into epithelial cells (18, 20, 22). Another mechanism for NTHi cell entry is endocytosis mediated by lipid rafts since lipid raft inhibitors were able to hinder NTHi invasion (23). Lipid rafts are cholesterol enriched areas on the epithelial cells plasma membrane that play pivotal roles in trafficking and signal transduction (24). Receptor mediated endocytosis or Clathrin mediated endocytosis was suggested as a possible way for the NTHi bacteria to invade airway epithelial cells and alveolar macrophages and evade the immune system by hiding in these cells (21, 25). NTHi infection has been shown to reduce E-cadherin, a protein required for tight junction formation and airway epithelial integrity (26). The mechanism in which NTHi reduces E-cadherin in airway epithelial cells is currently unknown. Nonetheless, NTHi-mediated reduction of E-cadherin in airway epithelial cells can lead to bacterial colonization at the basal lamina and result in perturbations of the airway epithelial cell barrier (27).

Recent studies have emphasized on the important role of NTHi-derived human IgA-protease B1 and B2 in cleaving human lysosomal-associated membrane protein 1 (LAMP1) that mediates NTHi survival inside the airway epithelial cells (17, 28). The mechanism by which LAMP1 cleavage results in NTHi survival within the airway epithelial cells needs to be further studied. Additionally, NTHi secreted IgA protease cleaves the human IgA_1_. IgA attaches to the bacteria and this attachment prevents binding of NTHi to the epithelial cells and inactivates the bacterial toxins (29). Remarkably, the secreted IgA levels in COPD and asthma patients are reduced compared to healthy individuals (30). By cleaving the remaining IgA in airways, NTHi can escape the immune surveillance of IgA to facilitate their survival within the cells.

By hiding in airway epithelial cells and undergo structural changes, secreting proteases and reducing host defense mediators, NTHi can cause repetitive and chronic infections of the lower respiratory tract.

## Evading Mechanisms by *Klebsiella Pneumoniae*

*Klebsiella pneumoniae* (Kp) is a Gram-negative bacterium most commonly known as a pulmonary pathogen (31). Kp infection has gained more attention due to its immunomodulatory effects and antibiotic resistance features. Interestingly, it has been shown in asthma and allergic inflammation environments, the lung Kp burden increases (32).

*Klebsiella pneumoniae* can overcome the human immune system and cause persistent infections in the lung. One mechanism employed by Kp to survive within the airways is to reduce airway neutrophil production of α-defensin, an antimicrobial peptide. Thijs et al. demonstrated that the nasal levels of α-defensins are significantly lower in asthma patients who are more susceptible to Kp infections (33). Furthermore, Kp hijacks airway cells by employing deubiquitinase cylindromatosis (CYLD) that is involved in NF-κB signaling inactivation (34, 35). Additionally, Kp can inhibit the production of inflammatory mediators and α-defensins from airway epithelial cells through inhibiting MAPK signaling pathway by upregulating MAPK phosphatase-1 (MKP-1) and activating nucleotide-binding oligomerization domain-containing protein 1 (NOD1). NOD1 is an intracellular PRR that responds to bacterial particles. MKP-1 and CYLD can synergistically inhibit production of neutrophil chemoattractant IL-8 from airway epithelial cells (36–38). Manipulating the immune system with these mechanisms can contribute to persistence of Kp infections in the lung. It has been shown that Kp-specific Outer membrane protein A (OmpA) favors an anti-inflammatory response in early stages of pneumonia (39). Fagundes et al. demonstrated that Kp infection in germfree mice was able to induce IL-10 production in the lung and inhibit the inflammatory response development by restricting production of pro-inflammatory mediators (40). Furthermore, Kp exploits the IL-10 production to attenuate the innate immune response through activation of its downstream target signal transducer and activator of transcription 3 (STAT3) (41). Interestingly, Greenberger et al. showed that neutralizing and inhibiting IL-10 was able to enhance the Kp clearance from the lung (42). To further support role of IL-10 in Kp infection, Dolgachey et al. showed that overexpression of IL-10 in the lung of Kp infected mice significantly increased bacterial survival and mouse mortalities (43).

In summary, Kp exploits several mechanisms to evade host immune system in order to persist in the lungs, which include inhibition of anti-inflammatory peptides, host defense inflammatory cytokines and chemokines, and promotion of anti-inflammatory cytokines.

## *Pseudomonas Aeruginosa* Impairs Human Airway Innate Immunity

*Pseudomonas aeruginosa* (Pa), is a Gram-negative opportunistic bacterium that causes acute infections in patients with CF, COPD, ventilator-associated pneumonia, and bronchiectasis (44). This facultative anaerobe bacterium is capable of adhering to human airway epithelial cells via their flagellum, pili, and other cell membrane components (45). Pa secretes several virulence factors that potentially sabotage innate immunity (45, 46).

In CF airway chronic Pa infection, the bacterium changes from an active moving form to a passive form by down-regulating its flagellin expression (47). Pa flagellin is one of the best known PAMPs that activate TLR5 signaling pathway through MyD88 and induce multiple pro-inflammatory cytokines (IL-8, IL-1ß) from airway epithelial cells and macrophages to recruit neutrophils and other inflammatory cells into the airways (48–50). Pa uses different strategies to escape the protective role of flagellin-mediated host immune response. Notably, Pa can secrete alkaline protease AprA and elastase LasB, which cleave the exogenous flagellin as a mechanism to evade flagellin mediated immune responses (47). Interestingly, degradation of flagellin monomers by AprA and LasB can reduce TLR5 activation induced inflammatory responses (47). In addition, Pa secretes other proteases including LasA and protease IV. These proteases interact with a wide range of molecules including the host and the bacteria as mentioned above, resulting in structural component and inflammatory mediator degradation and dampening the immune responses. Pa proteases have been shown to degrade a variety of host defense mediators and components including but not limited to IL-8, CXCL1, CXCL5, IFN-γ, IL-6, immunoglobulins, and antimicrobial peptides (51–54).

The effects of elastase LasB in modulating the immune response during Pa infections have been well studied. Pa exploits LasB to inhibit alveolar macrophages oxidative burst and down-regulate reactive oxygen species (ROS) generation during phagocytosis (55). LasB is also involved in degradation of surfactant protein SP-A resulting in phagocytosis resistance of the bacteria (56, 57). Another LasB dependent immune modulating function is lysis of the thrombin protein resulting in formation of the FYT21 peptide that inhibits the activation of transcription factors NF-κB and AP-1 (58). Remarkably, asthma patients have about 20% more thrombin concentration compared to healthy individuals (59).

An additional strategy developed by Pa in chronic infections is that it is capable of halting the expression of the type III secretion system (T3SS) (60). T3SS is a needle like secretion machinery complex in Gram-negative bacteria that injects bacterial effectors into the host cells (61). This machinery complex has been implicated in pathogenesis of acute Pa infections by causing tissue damage and bacterial spreading (62, 63). By inhibiting T3SS expression, bacteria escape T3SS mediated inflammasome activation by chronic Pa infection in CF patients (64).

Notably, Pa can form biofilms, a specific bacteria self-aggregates via their extracellular matrix to form a multicellular matrix (65). The host response to Pa biofilm is rather intricate since it can both incite or inhibit the immune response. It has been shown that during the biofilm stage, Pa down-regulates flagellin and T3SS resulting in lower complement system activation (66–68). On the other hand, biofilms can conceal the surface bacterial factors from the host and helping Pa to evade the immune system. In contrast, Pa biofilms can stimulate the neutrophilic response and furthermore cause necrotic killing of neutrophils leading to a further robust inflammation and tissue damage (69, 70).

Mucoid Pa, the most common in CF chronic infections, produces excess amount of extracellular polysaccharide alginate called mucoidy (71). It has been shown that over-production of alginate can impair the immune response and allow for persistence of the bacteria with different strategies. Alginate interferes with opsonic phagocytosis and compliment system activation and disrupts ROS production during phagocytosis (65, 72). Alginate is also responsible for bacterial resistance to antimicrobial peptides like LL-37 (73). Furthermore, it has been shown that due to co-regulation of alginate and flagellin, mucoidy can inhibit synthesis of flagellin and thus reduce TLR5 activation (74, 75).

Another adaptive mechanism in which Pa escapes the host immune system is synthetic and structural changes to LPS in chronic infections. LPS structure is recognized by TLR4 and is composed of three components: lipid A, core oligosaccharide, and the O antigen. The O antigen is the main component which shows high variability in immunogenic oligosaccharides and interacts with extracellular surroundings (76). During Pa chronic infection, the bacterial LPS undergoes adaptive structural and synthetic changes which results in lipid A modification/acetylation, loss of O antigen polysaccharide, and downregulation of LPS synthesis (76). These adaptive changes are considered to be possible mechanisms that Pa exploits to escape the immune system perhaps through being unrecognizable to TLR4 (77). Moreover, lipid A undergoes acetylation, which prevents binding of host antimicrobial peptides to the bacteria (78). It seems that Pa causing the chronic infection has more tendency to undergo genetic mutations resulting in significant lower expression to no expression of the O antigen involved in lower clearance of the bacteria from the lungs of CF patients (79–81).

Together, Pa uses various mechanisms to sustain lung chronic infections, such as reducing the recognition of flagellin by TLR5 and LPS by TLR4 through cleaving the TLR ligands, degrading host immune mediators or their transcription factors via secreting proteases, undergoing structural changes like forming biofilm, and producing excess amount of mucoid to evade host defense mechanisms and persist in the lungs.

## Crafty *Staphylococcus Aureus* Manipulates Host Immune Responses

*Staphylococcus aureus* (Sa) is a Gram-positive bacterium contributing to a range of diseases. Sa is a commensal of the human nose without showing any symptoms, but it can cause life-threatening diseases like pneumonia, endocarditis and septicemia (82). Sa is not an intracellular pathogen, but it can bind the epithelial and macrophage cell surface type F scavenger receptor SREC-I through the bacterial glycol-polymer cell wall teichoic acid (83). Furthermore, clumping factor B (ClfB) and iron-regulated surface determinant A (IsdA) are also involved in adherence of Sa to epithelial cells (84).

One of the mechanisms by which Sa can evade the immune system of the lung is to produce peptides that disturb accumulation of the complement system on the surface of the bacteria. Staphylococcal immunoglobulin binding protein (Sbi) depletes complement factor 3 (C3) (85). Moreover, the chemotaxis inhibitory protein of Sa (CHIPS) hinders the function of complement factor C5a. CHIPS also impedes the function of formylated peptide receptors on neutrophils that are required for neutrophil chemotaxis and recruitment to the site of infection (86).

There are multiple Sa proteases that can play a pivotal role in dampening the innate immune system. One of these proteases is Staphopain A, a serine protease, cleaves the N terminal domain of CXCR2, which inhibits the binding of CXCR2 to its ligand IL-8 and subsequently neutrophil chemotaxis and activation (87). Meanwhile, Staphopain B can cleave CD31, a member of immunoglobulin superfamily, expressed on neutrophils, and lead to reduced functionality of neutrophils (88). Besides, the Zn dependent metalloprotease, Aureolysin, can cleave and deactivate LL-37, an antimicrobial peptide (89). Another mechanism employed by Sa to dampen the immune system is by expressing a sortase anchored protein to dephosphorylate and activate adenosine (90). Adenosine is an effective mediator of the immune response and it binds to different G proteins coupled receptors. Upon binding adenosine to its receptor, the anti-inflammatory signaling pathways activate and result in inhibition of neutrophils degranulation and superoxide burst, platelet aggregation, and secretion of anti-inflammatory cytokine IL-10 from the innate immune cells (91).

Coagulation is the conversion of the fibrinogen to fibrin by activated thrombin and forming fibrin clots. Coagulation is one of the innate immunity defense mechanisms involved in immobilization of the bacteria and recruiting/activating the immune cells to the lungs to clear the bacteria by phagocytosis. Sa secretes coagulase (Coa) and von Willebrand Factor-binding protein (vWbp) which activate a prothrombin named staphylothrombin that only cleaves fibrinogen A and B peptides and creates fibrils of fibrin (92). Staphylothrombin avoids clotting activation and inflammation by only cleaving fibrinogen A and B but not other thrombin substrates (93, 94).

In summary, Sa utilizes a variety of elaborated mechanisms to evade the immune response including adhering to the epithelial cells and other airway innate immune cells, disrupting and depleting the complement system and neutrophil macrophages, cleaving and deactivating antimicrobial peptides, and activating adenosine mediated anti-inflammatory signaling pathways. These mechanisms may contribute to antibiotic resistance of Sa, and thus need to be further investigated for developing alternative therapies.

## Non-Tuberculous Mycobacteria Veil From the Host Immune System

Non-tuberculous mycobacterium (NTM) has emerged as a significant cause of infection in the lungs of immunocompromized patients or patients with CF, COPD, and bronchiectasis. Different from *Mycobacterium tuberculosis*, NTMs are opportunistic bacteria that can cause airways infection in altered lung environment specifically in chronic inflammatory lung diseases (95, 96). Interestingly, patients under corticosteroid therapies are more susceptible to NTM infections. The underlying mechanism of this susceptibility is unknown.

Non-tuberculous mycobacterium bacteria are composed of more than 170 species with various virulence potencies. Among these species, *Mycobacterium avium* and *Mycobacterium abscessus* are the most frequent causes of NTM related pulmonary diseases (95–100).

Wide spectrum antibiotic resistances of NTMs are well studied but these bacteria also benefit from a variety of mechanisms that they can exploit and evade the immune system.

Non-tuberculous mycobacterium can grow inside and outside of cells. *M. avium* and *M. abscessus* can reside within macrophages and hide from the immune system and anti-microbial mediators. *M. avium* can be discharged from macrophages and infect other macrophages and spread (101). In contrast, *M. abscessus* is able to deter phagocytosis by restricting intra-phagosomal acidification and as a result persist within macrophages (102, 103).

Defective cilia functionality and excess mucus in the lungs of patients with chronic inflammatory lung diseases allow persistence of the bacteria. It has been shown that *M. abscessus* can enter a slow growth phase and persist better in the mucus of CF patients (104, 105).

Another mechanism in which *M. avium* and *M. abscessus* escape the immune response is to form biofilms. NTMs can develop biofilms within thickened alveolar airways in CF patients or within the lung cavities of COPD patients (106, 107). The biofilm can create a shield to prevent antimicrobial mediators penetration and allow persistence of the bacteria in the lungs (108–111).

Interestingly, the smooth variant of *M. abscessus* expresses glycopeptidolipid (GPL), which has been shown to mask the bioactive, immunostimulatory cell wall lipids of the bacteria. This coverage results in becoming unrecognizable from the TLRs and inhibition of production of inflammatory mediators and antimicrobial peptides and the innate immune response (112).

Therefore, NTMs continue to pose a huge health concern especially in patients with compromised immunity as they undergo physiological adaptations and mechanisms in the lung, such as persisting within macrophages or deterring phagocytosis, entering a slow growth phase, and masking immunostimulatory cell wall components via GPL expression or biofilm formation.

## Conclusion and Perspectives

One of the pivotal responsibilities of the immune system is to induce an appropriate inflammatory response to colonizing pathogens. However, many of these pathogens exploit diseased environments and impaired defense mechanisms in the host to develop various strategies and genetic polymorphisms to either stimulate tolerance, escape the immune response, or manipulate the immune system to survive in the body. As mentioned above, in allergic airways, pathogenic bacteria either inactivate the TLRs to modulate the immune system to their benefit or become unrecognizable to the TLRs. One example is Pa in CF airways reducing its flagellin expression and evades the consequences of being recognized by TLR5 to induce inflammation. Reducing anti-microbial peptides and inflammatory mediators, increasing anti-inflammatory mediators, secreting proteases, hiding within the host cells, and structural changes are other mechanisms employed by bacteria in chronic inflammatory airway diseases resulting in reduced clearance of the pathogens and persistence of the bacterial infection in the lungs of these patients. Current studies are exploring the immune response evasion mechanisms of pathogenic bacteria in disease environments. Under the circumstances that host diseased environment can affect the pathogenicity and persistence of the bacteria in the lungs is a significant area of ongoing research.

In the future, the mechanisms behind differential regulation and functions of TLRs in healthy individuals vs. patients in various host environments should be considered for further investigation aimed to unravel this complex system. Moreover, the host and pathogen factors that help pathogens evolve very sophisticated strategies to evade the immune system and inflammation should be further determined. For example, how therapies like corticosteroids commonly used in chronic inflammatory diseases such as COPD and asthma affect bacterial evading mechanisms remain unclear, and could be carefully evaluated. Given significant growth of antibiotic resistant bacterial strains, new therapies targeting the host rather than the bacteria can be developed to allow the host to outsmart the pathogens.

## Author Contributions

Both authors listed have made a substantial, direct and intellectual contribution to the work, and approved it for publication.

## Conflict of Interest

The authors declare that the research was conducted in the absence of any commercial or financial relationships that could be construed as a potential conflict of interest.
